# CD9 role in proliferation, rejuvenation, and therapeutic applications

**DOI:** 10.1016/j.gendis.2023.05.009

**Published:** 2023-06-30

**Authors:** Sai Priyanka Kodam, Neda Baghban, Mujib Ullah

**Affiliations:** Institute for Immunity and Transplantation, Stem Cell Biology and Regenerative Medicine, School of Medicine, Molecular Medicine Department of Medicine, Stanford University, Palo Alto, CA 94304, USA

The CD9 gene, also known as the Tspan29 gene, codes for a protein called CD9, which is a member of the tetraspanin family of transmembrane proteins.^1^ The CD9 protein is involved in various cellular processes, including cell proliferation, differentiation, adhesion, and migration.^1^^,^^2^ CD9 has also been found to play a role in the regulation of stem cell proliferation and differentiation.^1^ Overall, the CD9 gene and its encoded protein play important roles in cellular processes related to proliferation, and further research may uncover new insights into its functions and potential therapeutic applications.^1^^,^^2^ CD9 is involved in a variety of cellular processes that occur at the plasma membrane, including cell adhesion, migration, and signaling.^1^ It has been shown to interact with other proteins in the plasma membrane, such as integrins, and to modulate their functions.^1^ CD9 also plays a role in the formation of tetraspanin-enriched microdomains or tetraspanin webs, which are specialized regions of the plasma membrane that are involved in signaling and membrane organization.^1^ In addition to its role in the plasma membrane of cells, CD9 has also been found in extracellular vesicles, including exosomes, which are released from cells and can play a role in intercellular communication.^3^ CD9 in exosomes has been shown to play a role in promoting cell adhesion, migration, and invasion in cancer cells.^2^^,^^3^ We overexpressed CD9 in induced pluripotent stem cells and observed high proliferation, enhanced pluripotency, and growth of stem cells. CD9 overexpression has been found to promote cell proliferation, migration, and invasion, as well as resistance to apoptosis.^1^^,^^4^ The effects of CD9 overexpression can vary depending on the specific cell type, the level and duration of overexpression, and other factors.^2^^,^^5^ Further research is needed to fully understand the mechanisms underlying the effects of CD9 overexpression and its potential therapeutic applications.

The extracellular domain of CD9 is the most variable and is involved in other tetraspanin synthesis and maturation.^1^ It has been shown that CD9 plays a role in exosome formation and secretion from the cells. Therefore, the CD9 overexpressed cells may lead to increased production of exosomes and their release.^1^^,^^4^

CD9 is one of several tetraspanin proteins that have been implicated in cell fusion.^4^ It has been found to be expressed in cells that undergo fusion, such as muscle cells and placental syncytiotrophoblasts.^2^^,^^4^ Studies have shown that CD9 is involved in the regulation of germ cell migration, adhesion, and survival during spermatogenesis.^4^ CD9 has been found to be an important component of exosomes, which are small extracellular vesicles that are released by many different cell types and can play a role in intercellular communication.^4^ Exosomes carry various bioactive molecules, including proteins, lipids, and nucleic acids, and they can be taken up by recipient cells, where they can modulate cellular processes.^4^ Studies have shown that CD9 expression levels in exosomes can be modulated by different stimuli, such as stress or signaling pathways, and that changes in CD9 levels can affect the biological activity of exosomes.^5^ CD9-enriched exosomes have been found to play a role in various physiological and pathological processes, such as immune response, cancer progression, and tissue repair.^1^

We overexpressed CD9 into human iPSCs and validated its expression by different assays. We performed the MTT assay which is a useful tool for assessing cell viability and proliferation. qPCR and Western blot analysis were used to analyze the gene and protein expression of CD9 (Fig. S1, 2). This analysis showed that this CD9 overexpression increased the expression of proliferation markers, pluripotent genes, and growth factors. One of the most important mechanisms through which this proliferation of CD9 was increased was the SIRT1-dependent pathway. Overexpression of CD9 resulted in overexpression of Ki67. Ki67 is a key proliferation marker that acts at protein and mRNA levels to up-regulate the expression of cell cycle protein^1^. SOX2, SSEA1, OCT4, and NANOG are transcription factors that play important roles in maintaining pluripotency and self-renewal in embryonic stem cells and induced pluripotent stem cells.^1^^,^^4^ It works by regulating the expression of genes involved in the maintenance of pluripotency, including OCT4 and SIRT1 (Fig. 1; Fig. S1, 2).

**Figure 1:**
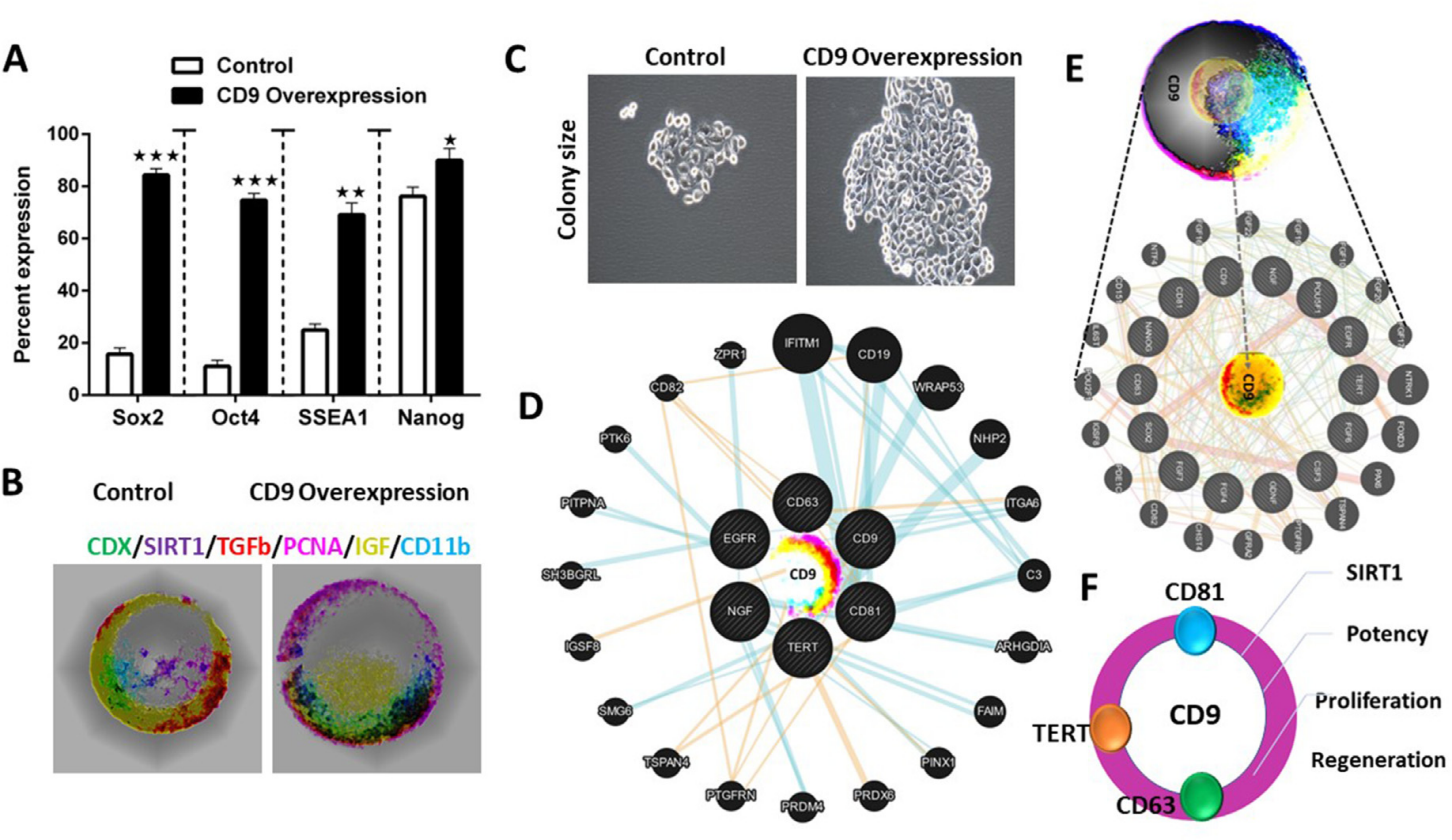
Role of CD9 overexpression in proliferation and bioinformatic analysis. **(A)** CD9 overexpression induced the expression of pluripotent genes (Sox 2, Oct 4, SSEA1, and Nanog). **(B)** The expression of PCNA and SIRT1 revealed enhanced proliferation in iPSCs. **(C)** CD9 overexpression resulted in increased colony size. **(D**–**F)** Bioinformatic analysis of CD9 overexpression in iPSCs revealed the interacting partners of CD81, CD63, and SIRT1. Gene functional analysis showed the association of CD9 with proliferation and regeneration via SIRT1. The data were expressed as the mean ± SD and analyzed by the *t*-test, ^∗^*P* < 0.05, ^∗∗^*P* < 0.01, and ^∗∗∗^*P* < 0.001.

CD9 has been shown to play a role in tissue regeneration.^1^ CD9 has been shown to promote the activation and proliferation of satellite cells, which can enhance muscle regeneration after injury.^1^^,^^4^ In liver regeneration, CD9 is expressed on the surface of hepatic progenitor cells, which can differentiate into mature liver cells to replace damaged tissue.^1^^,^^4^ CD9 has been shown to promote the migration and proliferation of hepatic progenitor cells, which can enhance liver regeneration after injury.^1^^,^^4^

The findings in our experiment strongly correlate with the previous hypothesis proposing the importance of CD9 in cell proliferation. What is unique about our study is that we explored the SIRT1 pathway. This pathway strongly suggested that SIRT1 positively regulates iPSC proliferation (Fig. 1). The interactions between CD9 and SIRT1 enabling this cell proliferation need to be further explored.^4^^,^^5^ It offers immense potential in upscaling the production of stem cells for stem-cell-based replacement therapies and transplants.^4^

SIRT1 is an NAD^+^-dependent deacetylase that is involved in the regulation of cellular metabolism, stress response, and aging.^4^ It has been shown to play a role in various physiological and pathological processes, such as DNA repair, inflammation, and cancer.^4^^,^^5^ SIRT1 can modulate the acetylation status of numerous target proteins, including histones and transcription factors, and thereby regulate gene expression.^4^ CD9 has been found to interact with SIRT1 and regulate its activity.^4^

In conclusion, there is convincing evidence to suggest CD9 up-regulates cell proliferation in iPSCs by increasing the growth factor expression.^4^ This property can be used in therapies to induce tissue repair and regeneration. Overall, the interaction between CD9 and SIRT1 is an area of active research, and further studies are needed to fully understand the mechanisms underlying their interaction and the physiological and pathological implications of this interaction.

## Conflict of interests

The authors declare no competing interests.
