# Supplementary material for: CD9 role in proliferation, rejuvenation, and therapeutic applications
Source: Genes Dis. 2023 Jun 30;11(3):101008. doi: 10.1016/j.gendis.2023.05.009 (PMC10825280; doi:10.1016/j.gendis.2023.05.009)
Supplement: Multimedia component 1 [file mmc1.docx]

**CD9 for cellular proliferation and rejuvenation**

CD9 is a heat shock protein and a transmembrane protein belonging to the tetraspanin family of proteins (1). Tetraspanin group of proteins are expressed ubiquitously in higher organisms. They are involved in multiple physiological processes like cell motility and adhesion, tumor invasion, fertilization, and virus infection (2-4). Human CD9 is a protein folded into four trans-membrane helices with the intracellular ends tightly bundled and the extracellular ends loosely packed, thus creating a transmembrane cavity. They possess a cone-like shape with short and long loops. (Figure 1) (5, 6). Multiple studies have suggested that the asymmetric shape and the clustering property of tetraspanins enable them to generate a curvature in the cell membrane facilitating reorganization of the membrane and modulating transmembrane protein distribution (5, 7). Depending on the cell type and interacting tissue components, they are involved in multiple processes such as cell-to-cell adhesion, interaction with extracellular space, cell signaling, migration, trafficking, apoptosis, inflammation, and immune response (8-10). These CD9 proteins control the membrane shape using specific mechanisms like insertion of amphipathic helices, insertion of hairpins and oligomerization into curved scaffolds. These mechanisms alter the sensitivity of membrane curvatures to target proteins (5, 11).

CD9 could interact with various factors of different tissues such as epidermal growth factor (EGF), pro-TGFα, receptor membrane-bound agonists and pro-amphiregulin to interfere in juxtacrine and paracrine signaling (12). Previous studies showed various biological effects of CD9 in cellular proliferation, migration and adhesion due to these complicated interactions of CD9 with tissue factors (10, 12). Some studies revealed that these differences in CD9 proliferative and migratory effects could be related to different types of cells, either (12, 13). Cancer cells such as colon, bladder, lung, pancreatic, squamous cell, and breast cancers with high CD9 expression showed to have a decrease in cellular proliferation and migration (14-20). However, in endothelial cells, Schwann cells and human adipose-derived mesenchymal stem cells, high CD9 expressed cells increased cellular proliferation and migration (12, 21, 22). It is supposed that the anti-proliferative effects of CD9 could be related to the Wnt signaling pathway (23). CD9 can accelerate tumorigenesis by promoting proliferation, migration and inhibiting apoptosis (24). Yet, human hepatocellular carcinoma cells overexpressing CD9 displayed reduced growth by suppressing NFkB, MAPK and AKT signaling pathways (25, 26). It is apparent that CD9 is, hitherto, a double-edge sword; involved in dual roles in promoting mammalian health and disease. However, the pathway of proliferative effects of CD9 marker is still unclear.

The proliferative effects of CD9 markers have been reported in some stem cells such as human bone marrow stromal cells (hBMSCs) and human adipose-derived mesenchymal stem cells (hAMSCs) (12). But no reports have been observed about the proliferative or anti-proliferative effects of CD9 on some stem cells such as induced pluripotent stem cells (iPSCs). The iPSCs are mature somatic cells that are reprogrammed and reached pluripotent states. Nowadays, they became a source for pre-clinical and clinical studies due to their potency for proliferation and differentiation (27, 28). CD9 effects cellular proliferation and migration, its exact role and pathway are still unclear. Considering the above, the current study was aimed to investigate the cytoprotective roles of CD9 as well as its role in the regulation of cell proliferation in iPSCs were evaluated.

## **Cloning of human CD9**

In order to encode the full‐length of human CD9 (hCD9) cDNA (GenBank accession no.: [AK056951](https://www.ncbi.nlm.nih.gov/nuccore/AK056951)) was amplified. For that, the following forward 5′‐GAGATATACATATGGAGATCC CTGTGC‐3′ and reverse 5′‐GTGCTCGAGTTACTTGGCTGCGGCTGGCGG‐3′ primers, were amplified with PCR amplification kit (System Biosciences). Then, the purification of the PCR result was performed in an agarose gel following the electrophoresis. It was then digested with XbaI and BamHI and inserted into the predigested plasmid vector pCDH. The copGFP expression was applied for verifying the construct. In the current study, the constructed Lentivirus (System Biosciences, USA) was a fourth-generation lentiviral vector modified with an insertion driving the combination of CMV promoter (driving the expression of CD9, Clontech, USA) and copGFP by P2A sequence.

## **Expression and purification of CD9 lentivirus**

The fourth-generation lentiviral vectors were generated in human embryonic kidney cells (HEK 293T/17). In detail, cells were cultured with DMEM (DMEM, Gibco, Life Technology Co., US) supplemented with 10% fetal bovine serum (FBS, Gibco, US) and 1% penicillin-streptomycin (Pen-Strep, Gibco, Life Technology Co., US) incubated at 37°C for 6 h. After that, the fresh medium was replaced and incubated for 72 h. After 2 days of transfection, a lentivirus-containing medium was collected. Then, the cellular debris was cleared using centrifugation at 1500×g for 5 min. The lentivirus particles were concentrated PEG-it virus precipitation solution (SBI, LV810A-1). The lentivirus particles pellet was resuspended with phosphate-buffered saline (PBS). At the final stage, the lentiviral particles were measured with the Lenti-X qRT-PCR Titration Kit (Clontech, USA).

## **Transfection of human induced pluripotent stem cells**

The human induced pluripotent stem cells (hiPSCs) were cultured in DMEM supplemented with 10% FBS and 1% Pen-Strep with seeding density of 1×10^5^ cells per well in triplicate in six-well plates. The cells were incubated for 36 h at 37 °C and 5% CO_2_. After that, the transfection was done by replacing the culture medium with 1 ml transfection medium including fresh medium supplemented with 100 μg/mL of sterile-filtered protamine sulfate (Sigma-Aldrich, MO, USA). The cells were infected with multiplicity of infection (MOI) 10, for 24 h, at 37 °C, and this phase was repeated with a MOI of 10 for a further 12 h for achieving the maximum transfection efficacy. Following 36 h and two rounds of transduction, the cells were trypsinized and plated at 500 cells/cm^2^ in T175 cm^2^ flasks in a fresh transfection medium as described previously. The fresh culture medium was changed three times a week. After expanding to about 70% confluency in approximately 5 days, the cells were lifted with trypsin/EDTA and continued to expand under the same conditions.

## **Measurement of Cell Viability and MTT assay**

Cellular proliferation was analyzed using the methyl tetrazolium (MTT) assay kit (Roche, USA). For MTT assay, the cells were seeded at density of 1×10^6^ cells/mL in a 96-well plate. Transfected and non-transfected cells were cultured in cell culture medium and seeded cells were incubated for 12 h. Then, MTT reagent was added to each well after diverse time points and incubated for 4 h until purple formazan crystal was formed. The DMSO was added to each well and it was incubated for 20 min. Five replicates were assayed for each experimental condition. The absorbance of formazan of cells was read by ELISA reader machine (BioTeck, USA). After each time, the viability assay was performed following the manufacturer’s instructions.

## **RNA isolation and qPCR**

To isolate RNA, each cell type (iPSCs, and CD9-transfected cells) was separately mixed with TriReagent (Sigma-Aldrich). Next, 1-bromo-3-chloropropane (Sigma-Aldrich) was added to all samples and centrifuged (45 min, 13,000 g). Then, the protein-free upper phase was collected and mixed with an equal volume of ethanol. The sample was then processed using the RNeasy Mini Kit (Qiagen, USA) according to the manufacturer’s recommendation. The quantity and quality of eluted RNA were measured using NanoDrop (NanoDrop Products, Thermo Fisher Scientific, Waltham, MA, United States).

For qPCR, the synthesis of cDNA was performed using 2.0 μg total RNA using the iScript cDNA Synthesis Kit (BioRad, USA). The TaqMan qPCR was performed three times in 96-well optical plates on the Applied Biosystem (Applied Biosystem, USA). The gene expression was assessed for all genes using TaqMan probes and primer sets (Applied Biosystems, USA). The analysis of the quantitative gene expression was performed for proliferating cell nuclear antigen (PCNA; [Hs00427214_g1](https://www.thermofisher.com/taqman-gene-expression/product/Hs00427214_g1?CID=&ICID=&subtype=)), marker of proliferation gene Ki-67 (MKi67; [Hs00606991_m1](https://www.thermofisher.com/taqman-gene-expression/product/Hs00606991_m1?CID=&ICID=&subtype=)), Sirtuin 1 (SIRT1; [Hs01009006_m1](https://www.thermofisher.com/taqman-gene-expression/product/Hs01009006_m1?CID=&ICID=&subtype=)) and glyceraldehyde-3-phosphate dehydrogenase (GAPDH; Hs99999905_m1). The expression of SIRT1, PCNA and MKi67 genes was normalized to the endogenous GAPDH expression level and calculated using the formula 2-ΔΔCt as a percentage of GAPDH expression.

## **Western blot analysis**

Western blotting was done in RIPA buffer containing protease inhibitor cocktail, 2.5 mM sodium pyrophosphate, 1 mM β-glycerolphosphate, 2 mM sodium vanadate, 1 mM EDTA, and 1 mM EGTA. Mouse aortas were homogenized in RIPA buffer (Sigma Aldrich, USA) and centrifuged (15,000 x g) for 15 min at 4℃. The protein concentration was measured using the Pierce BCA assay (Thermo Scientific). The protein (20 μg protein/well) under reduced conditions were directly subjected to SDS-PAGE (4–15% Tris-HCL precast gel, Bio-Rad)) was transferred onto nitrocellulose filters after separation. Blots were blocked in 5% BSA in Tris-buffered saline and Tween 20 for 1 hour, and the membranes were incubated overnight (4℃) with antibodies against SIRT1 (diluted 1:500, Cell Signaling Technology, USA), and β-actin (diluted 1:10,000, Abcam, USA).

## **Statistical analysis**

The SigmaStat 3.5 software (Systat Software, USA) and GraphPad Prism4 (GraphPad Software, Inc, San Diego. CA, USA) were applied for the statistical analysis and drawing graphs, respectively. The simple Student *t*-test was applied for statistical comparison of data and asterisks of *, **, and *** were assigned to statistically significant values of P< 0.05, P< 0.01, and P < 0.001, whereas exact P values were mentioned for statistically nonsignificant data sets. All the results were presented as mean ± standard error of the mean.

# **Transfecting induces pluripotent stem cells (iPSCs) with CD9-overexpressing construct.**

lentiviral vector construct, pLenti-CMV-CD9-copGFP (Fig. 2A), was constructed in which the expression of CD9 was driven by a CMV promoter. To track and monitor the expression of CD9 gene, it was linked to copGFP for easy detection of the transduction of target cells. A lentiviral vector construct, pLenti-CMV-copGFP, was used as an internal control. After packing the construct into lentiviral particles, iPSCs were transfected. The expression of CD9 transfected cells were validated through morphology (Fig. 2B) and higher expression of copGFP (Fig. 2C-D). Further the HSP protein was positive for CD9 specific antigens as shown by immunofluorescence ELISA (Fig. 2E) and western blot (Fig. 2F). CD9 overexpression was also authenticated by flow cytometry (Fig. 1G) and real time PCR (Fig. 1H). All these results confirm well documented expression of CD9.

Thus, CD9 upregulates cell proliferation in iPSCs and therefore may be beneficial in tissue repair therapies and regeneration.

**References**

1. Levy S, Shoham T. Protein-protein interactions in the tetraspanin web. Physiology. 2005.

2. Hemler ME. Tetraspanin functions and associated microdomains. Nature reviews Molecular cell biology. 2005;6(10):801-11.

3. Wang J-C, Bégin LR, Bérubé NG, Chevalier S, Aprikian AG, Gourdeau H, et al. Down-regulation of CD9 expression during prostate carcinoma progression is associated with CD9 mRNA modifications. Clinical cancer research. 2007;13(8):2354-61.

4. Mikuličić S, Fritzen A, Scheffer K, Strunk J, Cabañas C, Sperrhacke M, et al. Tetraspanin CD9 affects HPV16 infection by modulating ADAM17 activity and the ERK signalling pathway. Medical microbiology and immunology. 2020;209(4):461-71.

5. Umeda R, Satouh Y, Takemoto M, Nakada-Nakura Y, Liu K, Yokoyama T, et al. Structural insights into tetraspanin CD9 function. Nature communications. 2020;11(1):1-11.

6. Zimmerman B, Kelly B, McMillan BJ, Seegar TC, Dror RO, Kruse AC, et al. Crystal structure of a full-length human tetraspanin reveals a cholesterol-binding pocket. Cell. 2016;167(4):1041-51. e11.

7. Umeda R, Satouh Y, Takemoto M, Nakada-Nakura Y, Liu K, Yokoyama T, et al. Structural insights into tetraspanin CD9 function. Nature Communications. 2020;11(1):1606.

8. Powner D, Kopp PM, Monkley SJ, Critchley DR, Berditchevski F. Tetraspanin CD9 in cell migration. Biochemical Society Transactions. 2011;39(2):563-7.

9. Ovalle S, Gutiérrez‐López MD, Olmo N, Turnay J, Lizarbe MA, Majano P, et al. The tetraspanin CD9 inhibits the proliferation and tumorigenicity of human colon carcinoma cells. International journal of cancer. 2007;121(10):2140-52.

10. Reyes R, Cardeñes B, Machado-Pineda Y, Cabañas C. Tetraspanin CD9: a key regulator of cell adhesion in the immune system. Frontiers in immunology. 2018;9:863.

11. Fanaei M, Monk PN, Partridge LJ. The role of tetraspanins in fusion. Biochemical Society Transactions. 2011;39(2):524-8.

12. Kim YJ, Yu JM, Joo HJ, Kim HK, Cho HH, Bae YC, et al. Role of CD9 in proliferation and proangiogenic action of human adipose-derived mesenchymal stem cells. Pflügers Archiv-European Journal of Physiology. 2007;455(2):283-96.

13. Xing C, Xu W, Shi Y, Zhou B, Wu D, Liang B, et al. CD9 knockdown suppresses cell proliferation, adhesion, migration and invasion, while promoting apoptosis and the efficacy of chemotherapeutic drugs and imatinib in Ph+ ALL SUP‑B15 cells. Molecular Medicine Reports. 2020;22(4):2791-800.

14. Mhawech P, Herrmann F, Coassin M, Guillou L, Iselin CE. Motility‐related protein 1 (MRP‐1/CD9) expression in urothelial bladder carcinoma and its relation to tumor recurrence and progression. Cancer: Interdisciplinary International Journal of the American Cancer Society. 2003;98(8):1649-57.

15. Mori M, Mimori K, Shiraishi T, Haraguchi M, Ueo H, Barnard GF, et al. Motility related protein 1 (MRP1/CD9) expression in colon cancer. Clinical cancer research. 1998;4(6):1507-10.

16. Si Z, Hersey P. Expression of the neuroglandular antigen and analogues in melanoma. CD9 expression appears inversely related to metastatic potential of melanoma. International journal of cancer. 1993;54(1):37-43.

17. Higashiyama M, Taki T, Ieki Y, Adachi M, Huang C-l, Koh T, et al. Reduced motility related protein-1 (MRP-1/CD9) gene expression as a factor of poor prognosis in non-small cell lung cancer. Cancer research. 1995;55(24):6040-4.

18. Sho M, Adachi M, Taki T, Hashida H, Konishi T, Huang Cl, et al. Transmembrane 4 superfamily as a prognostic factor in pancreatic cancer. International journal of cancer. 1998;79(5):509-16.

19. Erovic BM, Pammer J, Hollemann D, Woegerbauer M, Geleff S, Fischer MB, et al. Motility‐related protein‐1/CD9 expression in head and neck squamous cell carcinoma. Head & Neck: Journal for the Sciences and Specialties of the Head and Neck. 2003;25(10):848-57.

20. Mhawech P, Dulguerov P, Tschanz E, Verdan C, Ares C, Allal AS. Motility-related protein-1 (MRP-1/CD9) expression can predict disease-free survival in patients with squamous cell carcinoma of the head and neck. British journal of cancer. 2004;90(2):471-5.

21. Ko E-M, Lee IY, Cheon IS, Kim J, Choi J-S, Hwang JY, et al. Monoclonal antibody to CD9 inhibits platelet-induced human endothelial cell proliferation. Molecules & Cells (Springer Science & Business Media BV). 2006;22(1).

22. Hadjiargyrou M, Patterson PH. An anti-CD9 monoclonal antibody promotes adhesion and induces proliferation of Schwann cells in vitro. Journal of Neuroscience. 1995;15(1):574-83.

23. Huang C-l, Liu D, Masuya D, Kameyama K, Nakashima T, Yokomise H, et al. MRP-1/CD9 gene transduction downregulates Wnt signal pathways. Oncogene. 2004;23(45):7475-83.

24. Chen S, Huang H, Yao J, Pan L, Ma H. Heat shock protein B6 potently increases non‑small cell lung cancer growth. Molecular medicine reports. 2014;10(2):677-82.

25. Matsushima‐Nishiwaki R, Adachi S, Yoshioka T, Yasuda E, Yamagishi Y, Matsuura J, et al. Suppression by heat shock protein 20 of hepatocellular carcinoma cell proliferation via inhibition of the mitogen‐activated protein kinases and AKT pathways. Journal of cellular biochemistry. 2011;112(11):3430-9.

26. Nagasawa T, Matsushima-Nishiwaki R, Yasuda E, Matsuura J, Toyoda H, Kaneoka Y, et al. Heat shock protein 20 (HSPB6) regulates TNF-α-induced intracellular signaling pathway in human hepatocellular carcinoma cells. Archives of biochemistry and biophysics. 2015;565:1-8.

27. Rowe RG, Daley GQ. Induced pluripotent stem cells in disease modelling and drug discovery. Nature Reviews Genetics. 2019;20(7):377-88.

28. Kouchakian MR, Baghban N, Moniri SF, Baghban M, Bakhshalizadeh S, Najafzadeh V, et al. The Clinical Trials of Mesenchymal Stromal Cells Therapy. Stem Cells International. 2021;2021:1634782.


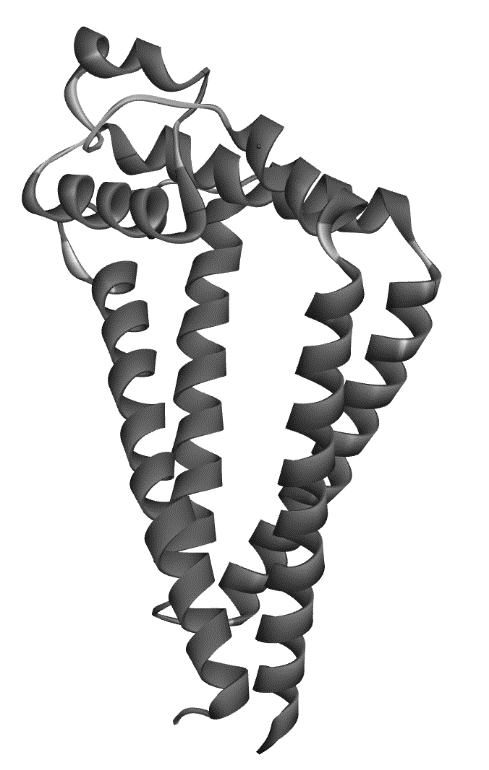


**Supplementary Figure 1** Reversed cone-like molecular shape of human CD9.


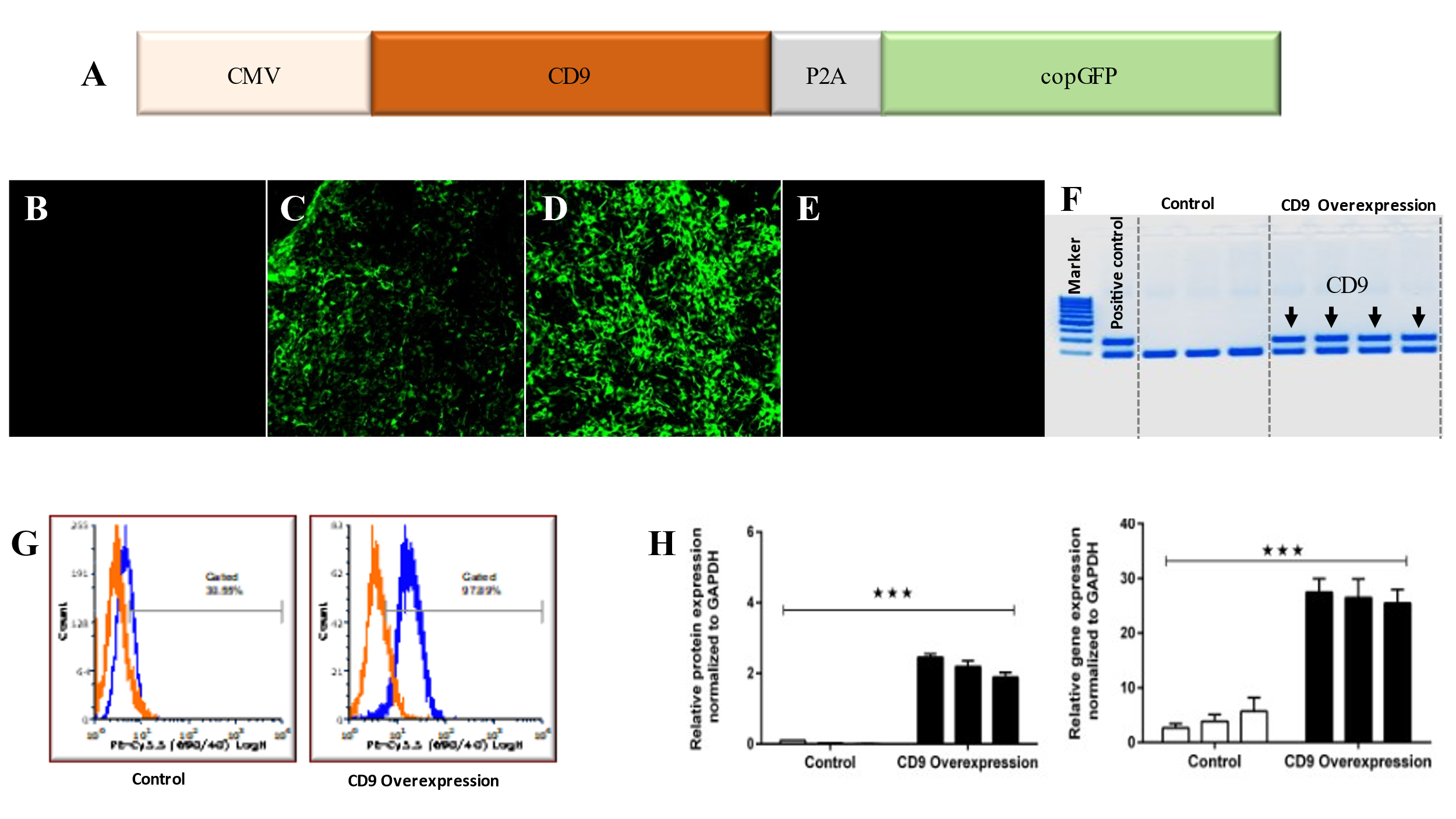


**Supplementary Figure 2-** Generation and molecular analysis of induced pluripotent stem cells (iPSCs) expressing CD9. A: Schematic diagram of the , pLenti-CMV-CD9-copGFP overexpression gene construct, in which the CD9 gene is under control of the a CMV promoter; B: Morphologic observation of induced pluripotent stem cells overexpressing CD9; C The positive expression of CD9 in iPSCs measured by expression of GFP; D: The positive expression of CD9 in iPSCs measured by expression of GFP; E: Fluorescence enzyme-linked immunosorbent assay showing the expression of CD9; F: Western blot showing the protein expression of CD9; G: Flow cytometry analysis confirming the expression of CD9; H: Real-time polymerase chain reaction analysis of the CD9. Data are expressed as the mean ± SD and were analyzed by the t-test, *P < 0.05, **P < 0.01, and ***P < 0.001.
